# Duration of Persistent Atrial Fibrillation Is Associated with Alterations in Human Gut Microbiota and Metabolic Phenotypes

**DOI:** 10.1128/mSystems.00422-19

**Published:** 2019-12-10

**Authors:** Kun Zuo, Jing Li, Pan Wang, Ye Liu, Zheng Liu, Xiandong Yin, Xiaoqing Liu, Xinchun Yang

**Affiliations:** a Heart Center & Beijing Key Laboratory of Hypertension, Beijing Chaoyang Hospital, Capital Medical University, Beijing, China; Mayo Clinic

**Keywords:** persistent atrial fibrillation, duration, gut microbiota, metabolism

## Abstract

Atrial fibrillation (AF) has been shown to be associated with disordered gut microbiota (GM). The underlying factors governing persistent AF (psAF) are not well understood, and the association between AF duration and GM profiles remains to be characterized. Thus, the present study aimed at investigating the dysbiosis of GM in patients with short and long psAF duration and illuminating the relationship between the GM and psAF maintenance. Based on metagenomic sequencing and metabolomic analyses, we assessed the metabolic and GM signature in 12 patients with psAF of <12 months (Pers<12m), eight patients with psAF of >12 months (Pers>12m), and 20 controls. We found that the GM in patients with both Pers<12m and Pers>12m was significantly perturbed, with an elevated microbial diversity, distinct structure, and discrepant composition. Although Pers<12m and Pers>12m patients shared a large number of common bacteria with controls, including 84 genera and 404 species, certain bacteria were differently enriched at different AF durations. Furthermore, disturbance in gut microbial function and GM-linked metabolic alterations were detected in both the Pers<12m and Pers>12m groups. The connection of GM and metabolites with psAF is consistent with interaction and potential modulation of host metabolic pathways due to GM dysbiosis with AF persistence. Our results showed that patients of the Pers<12m and Pers>12m groups shared many common disordered GM and metabolic features, which might occur in early disease, while prolonged psAF duration was related to certain unique alterations. Preventative strategies targeting GM and microbial metabolites for early intervention to treat AF patients are highly warranted.

**IMPORTANCE** Atrial fibrillation was associated with a disordered gut microbiota in previous research. However, the gut microbiota signature of patients at different stages of atrial fibrillation remains largely unknown. We sought to determine whether the shift in the gut microbiota and metabolic profiles occurs early and remains stable or develops gradually during atrial fibrillation. We found that patients with persistent atrial fibrillation of <12 months and persistent atrial fibrillation of >12 months shared most of the common features of gut microbiota dysbiosis. However, some distinctive and progressive alterations in the gut microbiota and metabolic structure, which may contribute to the progression of atrial fibrillation, were identified. The present study provides a comprehensive description of the dysbiotic gut microbiota and metabolic profiles in patients of short and long persistent atrial fibrillation, and our findings may help identify therapeutic strategies targeting the gut microbiota to treat atrial fibrillation at an early stage.

## Abstract (precis)

Atrial fibrillation was associated with a disordered gut microbiota in previous research. However, the gut microbiota signature of patients at different stages of atrial fibrillation remains largely unknown. We sought to determine whether the shift in the gut microbiota and metabolic profiles occurs early and remains stable or develops gradually during atrial fibrillation. We found that patients with persistent atrial fibrillation of <12 months and persistent atrial fibrillation of >12 months shared most of the common features of gut microbiota dysbiosis. However, some distinctive and progressive alterations in the gut microbiota and metabolic structure, which may contribute to the progression of atrial fibrillation, were identified. The present study provides a comprehensive description of the dysbiotic gut microbiota and metabolic profiles in patients of short and long persistent atrial fibrillation, and our findings may help identify therapeutic strategies targeting the gut microbiota to treat atrial fibrillation at an early stage.

## INTRODUCTION

Despite the application of medical and ablative therapy, atrial fibrillation (AF), one of the most prevalent and widespread arrhythmias, has remained a heavy global burden in the past decade and directly affects the quality of human life (1, 2). The rapid and irregular beating of the atria in AF is independently associated with a 2-fold- and 1.5-fold-increased risk in all-cause mortality in females and males, respectively, including stroke and heart failure (3–6). AF has been identified as a progressive disease, with an “AF begets AF” effect that fosters disease progression (7). Each episode of AF induces atrial electrical and structural remodeling, complex self-sustaining electrical activity, and even irreversible atrial fibrosis, which together contribute to the maintenance of AF (8, 9). Based on an electrocardiogram and AF duration, persistent AF (psAF) is divided into two clinically defined groups: (i) psAF of <12 months (Pers<12m) refers to a duration of AF disease for longer than 7 days but less than 1 year, and (ii) psAF of >12 months (Pers>12m) refers to a duration of AF of greater than 1 year (10). In general, Pers>12m is coupled with a sharp decline in AF termination rates and a greater number of individualized extrapulmonary vein drivers, which necessitates a wide variation in ablation therapies (11). However, the precise causes of AF persistence remain to be established. Uncovering the driving factors governing AF progression and identifying strategies to impede psAF have attracted considerable attention.

At the same time, emerging evidence has confirmed the involvement and important regulatory role of the gut microbiota (GM) in diverse diseases such as hypertension (HTN) (12, 13), type 2 diabetes mellitus (T2DM) (13, 14), obesity (15), coronary atherosclerotic heart disease (16–18), and heart failure (19). The underlying mechanism is attributed to an unbalanced immune response and complicated cross talk between GM metabolites and the target organ (20, 21). Owing to its involvement in such diseases, the association of the GM and alterations in metabolic patterns with AF was the focus of our previous research (22). Although we have revealed that a disordered GM and discrepant microbe-related metabolites are present in AF, extensive research is still needed to explore the clinical significance of the GM in AF progression.

To date, the GM and metabolic signature of patients at different stages of psAF remain unknown. We sought to determine whether a shift in the GM and metabolic profiles occurs early and remains stable or develops gradually and dynamically during AF progression and aggravation. To answer this question, we investigated whether patterns of dysbiotic GM are associated with the duration of psAF. To provide a comprehensive understanding of GM profiles throughout the duration of AF, we compared the GM and metabolic features of patients suffering from Pers<12m and Pers>12m based on metagenomic and metabolomic analyses, explored the alterations in GM diversity and structure, and analyzed the correlation between the GM and metabolites.

## RESULTS

### Baseline characteristics of the study cohort.

The study cohort included 40 participants consisting of 20 psAF patients and 20 controls (CTRs). According to clinical symptoms, results from electrocardiogram, duration of AF history, and degree of persistence, the 20 psAF patients were divided into 12 Pers<12m with psAF duration for less than 1 year and 8 Pers>12m with psAF duration for longer than 1 year. The clinical characteristics of our study cohort are shown in Table 1. The baseline clinical characteristics between Pers<12m and Pers>12m were similar, with no statistical differences in age, gender, body mass index (BMI), HTN diagnosis, diabetes mellitus diagnosis, or serum levels of total cholesterol, fasting blood glucose, creatinine, total bilirubin, or glutamic-pyruvic transaminase. Compared with the control group, Pers<12m and Pers>12m subjects were older with a higher BMI, higher incidence of T2DM, lower total cholesterol serum levels, and higher use of medications, including angiotensin-converting enzyme inhibitors (ACEI), angiotensin receptor blockers (ARB), amiodarone, statin, and dimethyl biguanide (DMBG).

**TABLE 1:** Baseline clinical characteristics of the study cohort^*a*^

Characteristic	Value by group:			*P* value		
	CTR	Pers<12m	Pers>12m	CTR vs Pers<12m	CTR vs Pers>12m	Pers<12m vs Pers>12m
No. of patients	20	12	8			
Age (yr), median (IQR)	53 (51, 55.75)	65 (58.5, 72)	68 (62.75, 71.75)	<0.001	<0.001	0.511
No. male/no. female	17/3	8/4	6/2	0.232	0.540	0.698
BMI, median (IQR)	24.50 (21.12, 27.41)	24.71 (22.85, 30.10)	28.79 (25.29, 33.16)	0.483	0.017	0.123
Other condition, no.						
HTN	11	5	6	0.472	0.336	0.152
DM	0	3	2	0.021	0.023	1.000
Blood chemistry, median (IQR)						
TC (mmol/liter)	4.73 (4.46, 5.25)	4.10 (3.50, 4.68)	4.25 (3.52, 5.10)	0.01	0.084	0.758
FBG (mmol/liter)	5.26 (4.85, 5.62)	5.14 (4.00, 6.48)	4.70 (4.47, 5.27)	0.799	0.052	0.440
Creatinine (μmol/liter)	69 (58.5, 95.68)	69.15 (64.05, 76.95)	70.45 (61.58, 76.53)	0.876	0.959	1.000
TBil (μmol/liter)	14.5 (11.57, 22.2)	21.45 (13.85, 25.1)	16.20 (12.95, 21.98)	0.096	0.690	0.334
ALT (U/liter)	22.5 (12.5, 29.25)	21 (12, 28)	24.5 (12.25, 44.25)	0.984	0.647	0.642
Medication, no. of patients						
ACEI	0	0	3			
ARB	0	1	1			
Amiodarone	0	1	2			
Statin	0	0	1			
DMBG	0	2	1			
psAF duration (mo), mean ± SD		1.34 ± 0.85	61.5 ± 67.71			<0.001
CHA2DS2-VASc score, mean ± SD	0.7 ± 0.571	2.42 ± 1.73	2.88 ± 1.73	0.006	0.003	0.571

a Abbreviations: CTR, control; psAF, persistent atrial fibrillation; Pers<12m, persistent atrial fibrillation of <12 months; Pers>12m, persistent atrial fibrillation of >12 months; BMI, body mass index; HTN, hypertension; DM, diabetes mellitus; TC, total cholesterol; FBG, fasting blood glucose; TBil, total bilirubin; ALT, glutamic-pyruvic transaminase; ACEI, angiotensin-converting enzyme inhibitors; ARB, angiotensin receptor blockers; DMBG, dimethyl biguanide; CHA2DS2-VASc score, congestive heart failure, hypertension, age of ≥75 years, diabetes mellitus, stroke/transient ischemic attack, vascular disease, age 65 to 74 years, sex category of female; IQR, interquartile range.

### Changes in gut microbiome diversity in patients with Pers<12m and Pers>12m.

**FIG 1:**
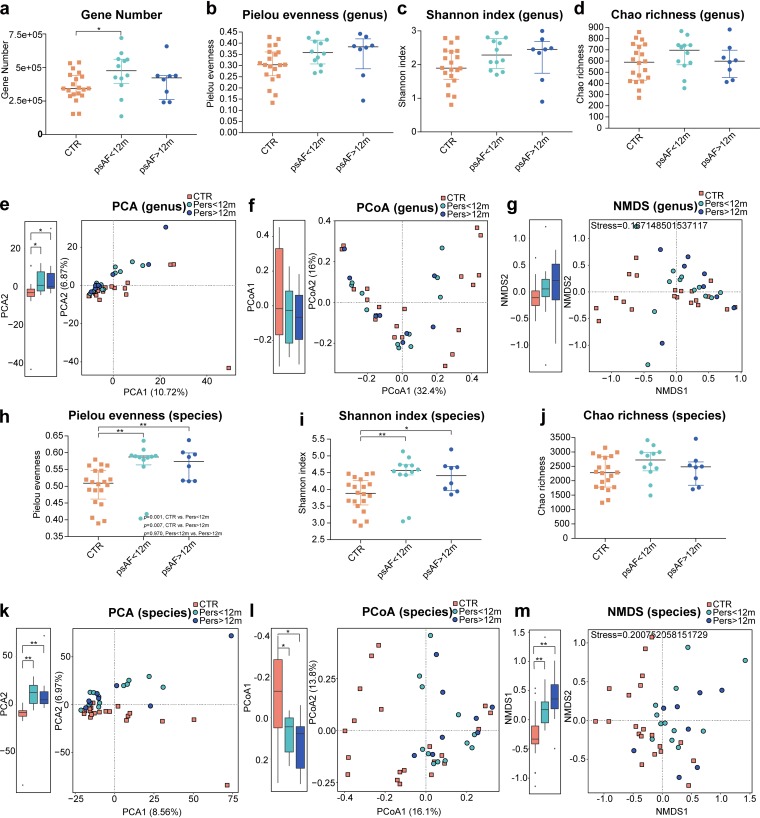
Changes in the gut microbiome diversity in Pers**<**12m and Pers>12m. Gene number (a) and alpha diversity including Pielou evenness (b and h), Shannon index (c and i), and Chao richness (d and j) based on the genus (b to d)/species (h to j) profile in the Pers<12m, Pers>12m, and CTR cohorts. The dots indicate individual values of the subjects, and the horizontal lines from bottom to top represent 25th percentiles, medians, and 75th percentiles, respectively. Beta diversity including principal-component analysis (PCA) (e and k), principal-coordinate analysis (PCoA) (f and l), and nonmetric dimensional scaling (NMDS) (g and m) based on abundances of the genera/species showed that the structures of GM in psAF of short and of long duration were similar to each other but distinct from controls. The orange squares represent CTR, green circles represent Pers<12m, and blue circles represent Pers>12m.

GM diversity reflects the number and variety of microbes in the gut and is often associated with disease states (17, 23–25). The total number of genes (Fig. 1a); the within-individual (alpha) diversity including Pielou evenness (Fig. 1b and h), Shannon index (Fig. 1c and i), and Chao richness (Fig. 1d and j); and the between-individual (beta) diversity including principal-component analysis (PCA) (Fig. 1e and k), principal-coordinate analysis (PCoA) (Fig. 1f and l), and nonmetric dimensional scaling (NMDS) (Fig. 1g and m), analyses based on either genus or species level, were used to assess the GM diversity in patients with Pers<12m and Pers>12m. We found that all parameters of GM diversity, regardless of genus or species level, showed similar trends in Pers<12m and Pers>12m compared to controls, and the scatter plots of PCA, PCoA, and NMDS based on GM abundances of bacteria of certain genus and species levels failed to distinguish psAF patients based on AF duration, indicating similar GM structures between Pers<12m and Pers>12m. Although no significant discrepancy in GM diversity was identified base on psAF of short or long duration, there was a dramatic difference in alpha and beta diversity between psAF patients and controls, suggesting that psAF patients develop GM dysbiosis at an early stage. The elevated diversity in the GM of psAF patients compared with controls indicates the possible overgrowth of a variety of harmful microbes.

Considering the difference in baseline characteristics, including age, BMI, T2DM diagnosis, total cholesterol levels, and medication use, between psAF and controls, we used PCA to assess whether the alterations in the GM observed in Pers<12m and Pers>12m patients were affected by these baseline factors (14, 26). Our results showed that the scatter in the plots was mixed and dispersed rather than clustered into separate groups, indicating the marginal impact of age, BMI, T2DM, total cholesterol, or medication on our findings (see Fig. S1 in the supplemental material).

10.1128/mSystems.00422-19.1 FIG S1 Influence of baseline characteristics, including age, sex, T2DM, total cholesterol, and medication use, on GM shifts. (a) PCA plot based on age and abundance of microbes at the genus level. Forty samples were divided into three groups according to age, <55 in yellow, 55 to 65 in light pink, and >65 in violet. Squares represent CTR, and circles denote psAF. (b) PCA plot based on body mass index (BMI) and abundance of microbes at the genus level. Forty samples were divided into 3 groups according to BMI, 18.5 ≤ BMI < 24 in blue, 24 ≤ BMI < 28 in orange, and BMI ≥ 28 in dark pink. Squares represent CTR, and circles denote psAF. (c) PCA plot based on type 2 diabetes mellitus and abundance of microbes at the genus level. Forty samples were divided into 2 groups according to T2DM history, those without T2DM in gray and those with T2DM in dark purple. Squares represent CTR, and circles denote psAF. (d) PCA plot based on TC and abundance of microbes at the genus level. One hundred samples were divided into 2 groups according to TC level, with TC < 5.18 in green) and TC ≥ 5.18 in pink. Squares represent CTR, and circles denote psAF. (e) PCA plot based on medication and abundance of microbes at the genus level. Twenty AF samples were divided into 6 groups according to their medication. Yellow triangles denote subjects with angiotensin-converting enzyme inhibitors (ACEI), inverted green triangles denote subjects with angiotensin receptor blockers (ARB), blue rhombi denote subjects with amiodarone, red circles denote subjects with dimethyl biguanide (DMBG), green asterisks denote subjects with statin, and gray squares denote subjects without medication. Download FIG S1, PDF file, 0.3 MB. Copyright © 2019 Zuo et al. 2019 Zuo et al. This content is distributed under the terms of the Creative Commons Attribution 4.0 International license.

### Dynamically altered GM community structure from Pers<12m to Pers>12m.

The microbial enterotype was examined to investigate whether the GM community structure underwent a shift in psAF from short to long duration. By the partitioning-around-medoids clustering methods based on the Jensen-Shannon divergence, the 40 samples were separated into two clusters (Fig. S2a) including an enterotype dominated by *Bacteroides* and an enterotype dominated by *Prevotella*, which have been previously reported in HTN, T2DM, and other diseases (12, 14, 27). Interestingly, a dynamic and progressive change in enterotype, with a transition from the *Prevotella* enterotype to *Bacteroides*, was observed. The distribution of control samples across enterotypes was relatively even, with 60% enterotype *Bacteroides* and 40% enterotype *Prevotella*, whereas the percentage of samples with enterotype *Bacteroides* was greater according to the duration of psAF (75% in Pers<12m, 87.5% in Pers>12m, *P* = 0.47, Fisher’s exact test [Fig. S2b and c]).

10.1128/mSystems.00422-19.2 FIG S2 Altered enterotype distribution in Pers<12m and Pers>12m. (a) Forty samples are clustered into enterotype *Prevotella* (red) and enterotype *Bacteroides* (blue) by principal-component analysis of Jensen-Shannon divergence values at the genus level. (b and c) The percentage of control Pers<12m and Pers>12m samples distributed in enterotype *Prevotella* and enterotype *Bacteroides*. A dynamic and progressive change of enterotype distribution, with transition from *Prevotella* enterotype to *Bacteroides*, was observed. Sixty percent CTRs, 75% Pers<12m, and 87.5% Pers>12m in enterotype *Bacteroides*, *P* = 0.47, Fisher’s exact test. Download FIG S2, PDF file, 0.4 MB. Copyright © 2019 Zuo et al. 2019 Zuo et al. This content is distributed under the terms of the Creative Commons Attribution 4.0 International license.

**FIG 2:**
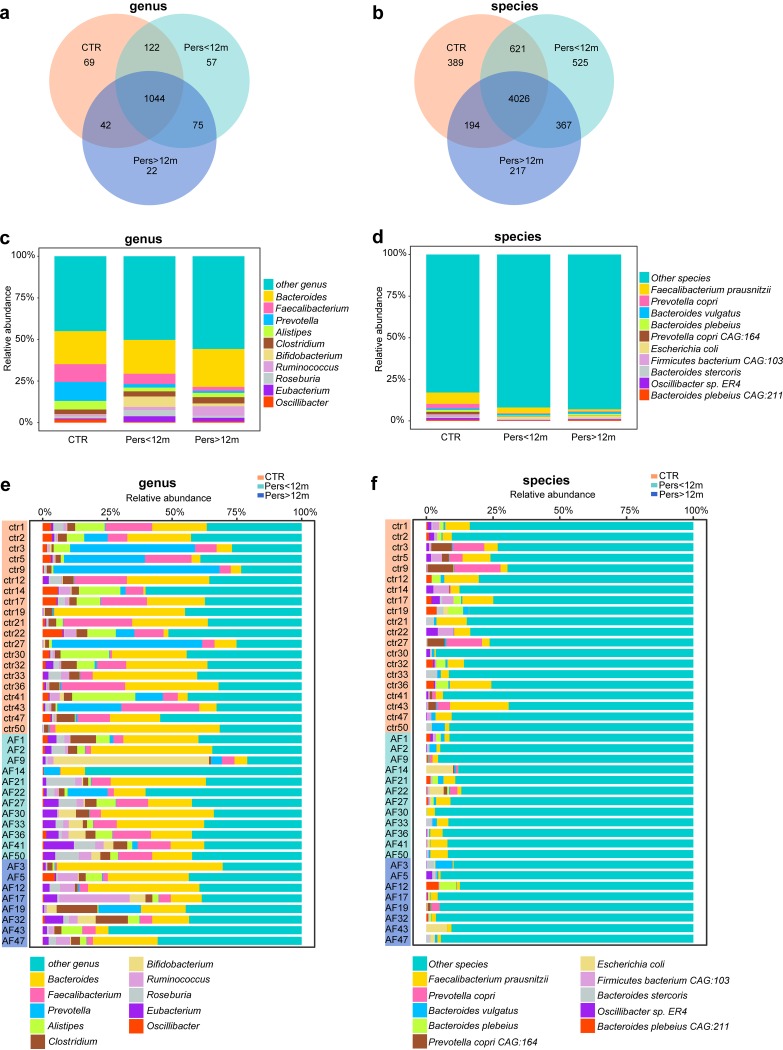
Dynamically altered GM community structure from Pers<12m to Pers>12m. (a) Venn diagrams demonstrating the number of annotated genera shared between CTR (orange), Pers<12m (green), and Pers>12m (blue). The overlap shows that there were 1,044 genera concurrently identified in CTR and psAF of short or long duration. (b) Venn diagrams demonstrating the number of annotated species shared among CTR (orange), Pers<12m (green), and Pers>12m (blue). The overlap shows that there were 4,026 species concurrently identified in CTR and psAF of short or long duration. (c) Bar plot of relative abundance of top 10 genera annotated in CTR, Pers<12m, and Pers>12m, where different genera are differentiated by color. (d) Bar plot of relative abundance of top 10 species annotated in CTR, Pers<12m, and Pers>12m, where different species are differentiated by color. (e) Bar plot of relative abundance of top 10 genera annotated in individuals from CTR (orange), Pers<12m (green), and Pers>12m (blue), where different genera are differentiated by color. (f) Bar plot of relative abundance of top 10 species annotated in individuals from CTR (orange), Pers<12m (green), and Pers>12m (blue), where different species are differentiated by color.

Additionally, we compared phylogenetic signatures from the gut of psAF patients in order to examine differences in GM composition with more specificity (Tables S1 and S2). Overall, Pers<12m and Pers>12m shared the vast majority of microbes annotated in this cohort, including 1,044 genera (Fig. 2a) and 4,026 species (Fig. 2b). The top 10 most abundant genera, such as *Bacteroides*, *Faecalibacterium*, and *Prevotella*, and species, such as Faecalibacterium prausnitzii, Prevotella copri, and Bacteroides vulgatus, in Pers<12m and Pers>12m exhibited remarkably distinct abundance compared with controls (Fig. 2c to f). Interestingly, the abundance of the top 10 genera or species showed a dynamic and progressive change, including an increase in *Bacteroides* and *Ruminococcus* and a decrease in Faecalibacterium prausnitzii, with the longer duration of psAF (Fig. 2c to f). This progressive change in the GM associated with the duration of psAF reveals a dynamic and aggravating profile of GM dysbiosis as the patients progresses to longstanding psAF.

10.1128/mSystems.00422-19.5 TABLE S1 Taxonomic annotation and abundance profiling at genus level. Download Table S1, XLSX file, 0.4 MB. Copyright © 2019 Zuo et al. 2019 Zuo et al. This content is distributed under the terms of the Creative Commons Attribution 4.0 International license.

10.1128/mSystems.00422-19.6 TABLE S2 Taxonomic annotation and abundance profiling at species level. Download Table S2, XLSX file, 1.9 MB. Copyright © 2019 Zuo et al. 2019 Zuo et al. This content is distributed under the terms of the Creative Commons Attribution 4.0 International license.

### Common and distinctive altered taxa in patients with Pers<12m and Pers>12m.

**FIG 3:**
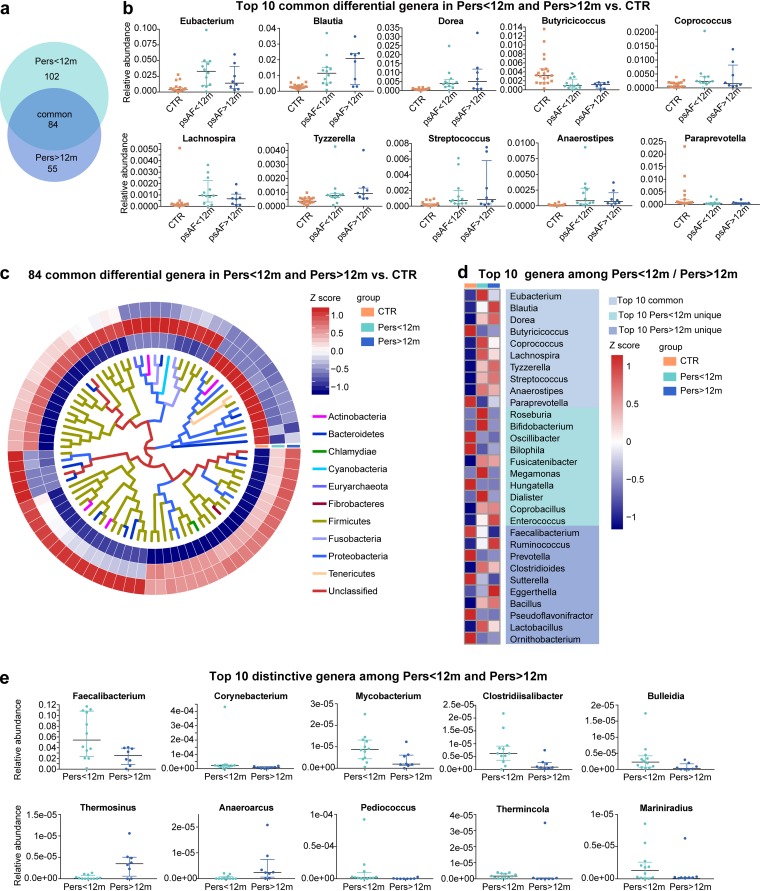
Common and distinctive genera in Pers<12m and Pers>12m. (a) Venn diagrams demonstrating the number of differential genera shared between Pers<12m (green) and Pers>12m (blue) compared with CTR (orange). The overlap shows that there were 84 genera concurrently identified in psAF of short or long duration. (b) Scatter plot of top 10 common differential genera in psAF of short or long duration. The dots indicate individual values of the subjects, and the horizontal lines from bottom to top represent 25th percentiles, medians, and 75th percentiles, respectively. (c) Heat map tree showing the 84 common differential genera in individuals from Pers<12m and Pers>12m groups compared with CTR at the criterion of *q* value of <0.05 (Wilcoxon rank sum test) and their phylogenic relationships. The abundance profiles are expressed by Z scores, and genera were clustered based on Bray-Curtis distance in the clustering tree. Z score is negative (shown in blue) when the row abundance is lower than the mean and shown in red when the row abundance is higher than the mean. The color of the inner lines denotes the phylum of certain genera. (d) Heat map of relative abundance of the top 10 common, Pers<12m unique, and Pers>12m unique genera at the criterion of *q* value of <0.05 (Wilcoxon rank sum test). The abundance profiles are transformed into Z scores by subtracting the average abundance and dividing the standard deviation of all samples. Z score is negative (shown in blue) when the row abundance is lower than the mean and shown in red when the row abundance is higher than the mean. (e) Scatter plot of top 10 distinctive genera between Pers<12m (green) and Pers>12m (blue). The dots indicate individual values of the subjects, and the horizontal lines from bottom to top represent 25th percentiles, medians, and 75th percentiles, respectively.

**FIG 4:**
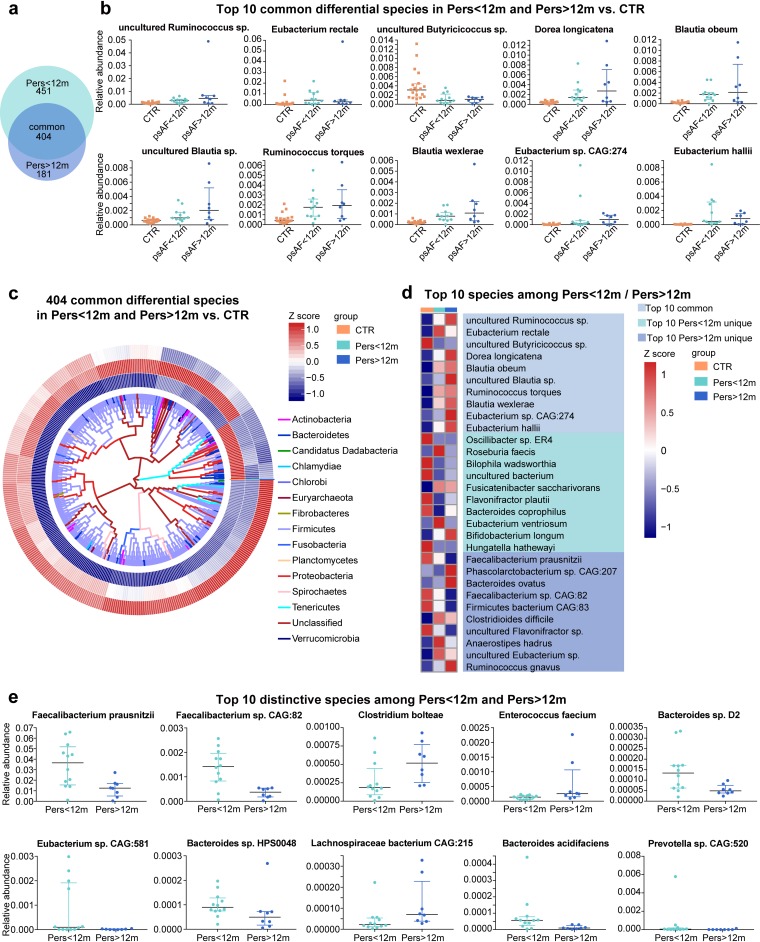
Common and distinctive altered species in Pers<12m and Pers>12m. (a) Venn diagrams demonstrating the number of differential species shared between Pers<12m (green) and Pers>12m (blue) compared with CTR. The overlap shows that there were 404 species concurrently identified in psAF of short or long duration. (b) Scatter plot of top 10 common differential species in psAF of short or long duration. The dots indicate individual values of the subjects, and the horizontal lines from bottom to top represent 25th percentiles, medians, and 75th percentiles, respectively. (c) Heat map tree shows the 404 common differential species in individuals from Pers<12m and Pers>12m group compared with CTR at the criterion of *q* value of <0.05 (Wilcoxon rank sum test) and their phylogenic relationships. The abundance profiles are expressed by Z scores, and species were clustered based on Bray-Curtis distance in the clustering tree. Z score is negative (shown in blue) when the row abundance is lower than the mean and shown in red when the row abundance is higher than the mean. The color of the inner lines denotes the phylum of certain genera. (d) Heat map of relative abundance of the top 10 common, Pers<12m unique, and Pers>12m unique species at the criterion of *q* value of <0.05 (Wilcoxon rank sum test). The abundance profiles are transformed into Z scores by subtracting the average abundance and dividing the standard deviation of all samples. Z score is negative (shown in blue) when the row abundance is lower than the mean and shown in red when the row abundance is higher than the mean. (e) Scatter plot of top 10 distinctive species between Pers<12m (green) and Pers>12m (blue). The dots indicate individual values of the subjects, and the horizontal lines from bottom to top represent 25th percentiles, medians, and 75th percentiles, respectively.

Subsequently, we analyzed the genera and species that were dramatically different between control and psAF subjects (*P* < 0.05; *P* values were tested using the Wilcoxon rank sum test and corrected for multiple testing with the Benjamin and Hochberg method). Compared with controls, 186 genera and 855 species were statistically different in Pers<12m, and 130 genera and 585 species were statistically different in Pers>12m (Table S3). Notably, Pers<12m and Pers>12m shared 84 common genera and 404 species (Fig. 3 and 4a), and these shared bacteria exhibited trends according to psAF duration. For example, genera such as *Butyricicoccus* and *Paraprevotella* showed a decreased trend with longer psAF duration, while genera such as *Blautia*, *Dorea*, and *Coprococcus* exhibited an increased trend with longer psAF duration (Fig. 3 and 4b to d). *Butyricicoccus* bacteria have been identified as short-chain-fatty-acid (SCFA)-producing bacteria, which may contribute to their beneficial effects for the host (28), while *Coprococcus* abundance has been associated with a greater risk for developing coronary heart disease in persons with chronic schizophrenia (29). Therefore, the decrease in beneficial microbes and/or enrichment in pathogenic microbes may be related to the pathology of psAF.

10.1128/mSystems.00422-19.7 TABLE S3 Detailed information on differential genera and species. Download Table S3, XLSX file, 0.10 MB. Copyright © 2019 Zuo et al. 2019 Zuo et al. This content is distributed under the terms of the Creative Commons Attribution 4.0 International license.

In addition to the shift in common taxa in psAF regardless of duration, we identified some distinctive changes in bacteria that were altered uniquely in Pers<12m or Pers>12m. Overall, 29 genera and 111 species were significantly different between Pers<12m and Pers>12m (Fig. 3 and 4e). Bacteria like *Thermosinus*, *Anaeroarcus*, Clostridium bolteae, and Enterococcus faecium were enriched in Pers>12m. Genera such as *Faecalibacterium* and *Corynebacterium*, and species like Faecalibacterium prausnitzii and *Eubacterium* sp. CAG 581 dominated in Pers<12m. We speculate that these common features of the GM may be linked to AF onset, and the unique shifts of the GM in Pers<12m or Pers>12m might account for the progression and persistence of AF disease.

### Microbial functions in patients with Pers<12m and Pers>12m.

**FIG 5:**
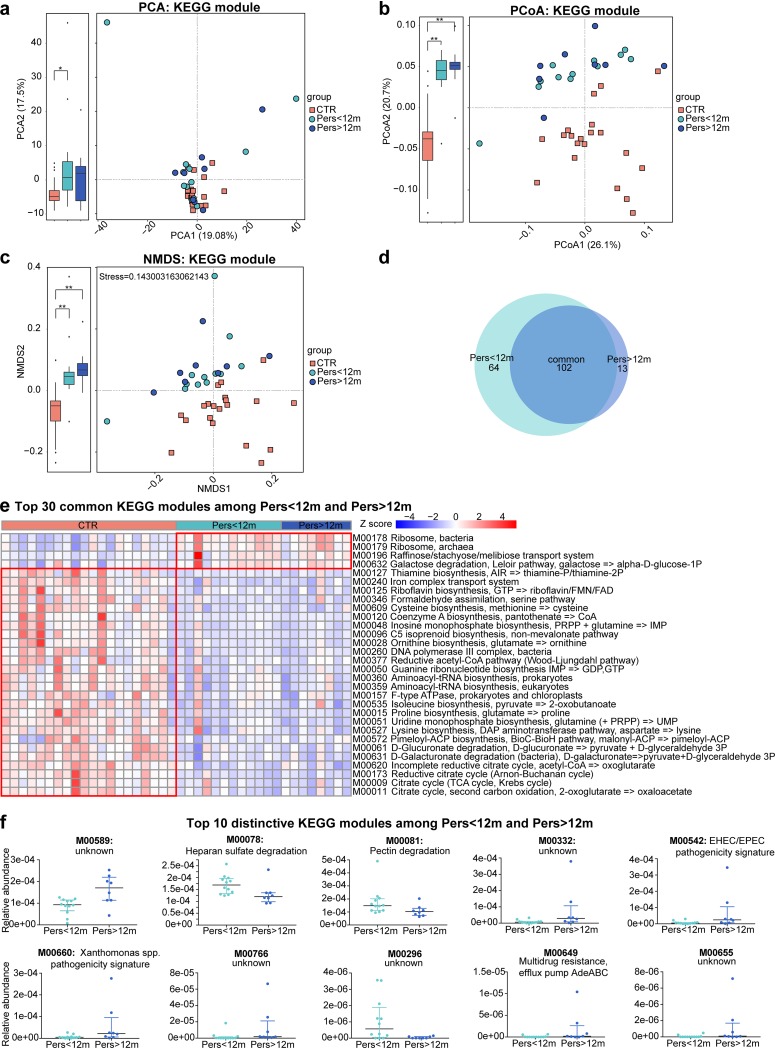
Microbial functions in Pers<12m and Pers>12m. (a to c) PCA (a), PCoA (b), and NMDS (c) based on abundances of the KEGG module showed the functions of GM in psAF of short or long duration were similar to each other but discriminative from controls. Orange squares represent CTR, green circles represent Pers<12m, and blue circles represent Pers>12m. (d) Venn diagrams demonstrating the number of differential functional modules shared between Pers<12m (green) and Pers>12m (blue) compared with CTR. The overlap shows that there were 102 KEGG modules concurrently identified in psAF of short or long duration. (e) Heat map of relative abundance of the top 30 common, Pers<12m unique, and Pers>12m functional modules at the criterion of *q* value of <0.05 (Wilcoxon rank sum test). The abundance profiles are transformed into Z scores by subtracting the average abundance and dividing the standard deviation of all samples. Z score is negative (shown in blue) when the row abundance is lower than the mean and shown in red when the row abundance is higher than the mean. (f) Scatter plot of top 10 distinctive KEGG modules between Pers<12m (green) and Pers>12m (blue). The dots indicate individual values of the subjects, and the horizontal lines from bottom to top represent 25th percentiles, medians, and 75th percentiles, respectively.

The KEGG databases were used to annotate the gut microbial gene functions as described previously (30). Using PCA, PCoA, and NMDS plots, the psAF and control subjects could be separated clearly, whereas Pers<12m and Pers>12m groups could not be distinguished from each other, suggesting significantly different microbial functions between psAF patients and controls but a similar pattern between Pers<12m and Pers>12m (Fig. 5a to c). There were 102 differentially enriched KEGG modules shared between Pers<12m and Pers>12m compared with controls (adjusted *P* < 0.05, Wilcoxon rank sum test [Fig. 5d and Table S4]). Interestingly, most of these shared modules also shared a similar variation tendency in Pers<12m and Pers>12m (Fig. 5e). The majority of the modules that were reduced in the psAF group are necessary for human health, such as aminoacyl-tRNA biosynthesis, the citric acid cycle, and iron complex transport, and the relative abundance of some of these metabolic functions are known to be decreased in patients with HTN, CHF, or liver cirrhosis (12, 23, 31). Moreover, 11 KEGG modules, such as enterohemorrhagic Escherichia coli (EHEC)/enteropathogenic E. coli (EPEC) pathogenicity signature, *Xanthomonas* pathogenicity signature, and multidrug resistance efflux pump AdeABC, were significantly elevated in the Pers>12m group (Fig. 5f). The specific relationship of these microbial functions in psAF progression remains to be elucidated.

10.1128/mSystems.00422-19.8 TABLE S4 Detailed information on differential KEGG modules. Download Table S4, XLSX file, 0.03 MB. Copyright © 2019 Zuo et al. 2019 Zuo et al. This content is distributed under the terms of the Creative Commons Attribution 4.0 International license.

### Alterations in the serum and gut metabolomics in psAF.

The underlying mechanisms whereby GM mediates human health depend on cross talk between GM metabolites and target organs. Thus, serum and fecal samples were analyzed by liquid chromatography-mass spectrometry (LC-MS) in both positive ion mode (electrospray positive [ES^+^]) and negative ion mode (ES^−^) to explore whether host metabolic pattern alterations were linked with dysbiotic GM during psAF progression. A subset of 29 participants (including 15 controls and 7 Pers<12m, and 7 Pers>12m patients) from the present study were enrolled in the serum metabolic study, and 26 individuals (including 9 controls and 10 Pers<12m and 7 Pers>12m patients) were enrolled in the feces study (Table S5). For serum, 2,540 features at ES^+^ ion mode and 1,173 features at ES^−^ ion mode were detected. For feces, 2,800 features at ES^+^ ion mode and 1,262 features at ES^−^ ion mode were tested in the present study.

10.1128/mSystems.00422-19.9 TABLE S5 Clinical characteristics of participants in serum and fecal metabolism studies. Download Table S5, DOCX file, 0.02 MB. Copyright © 2019 Zuo et al. 2019 Zuo et al. This content is distributed under the terms of the Creative Commons Attribution 4.0 International license.

**FIG 6:**
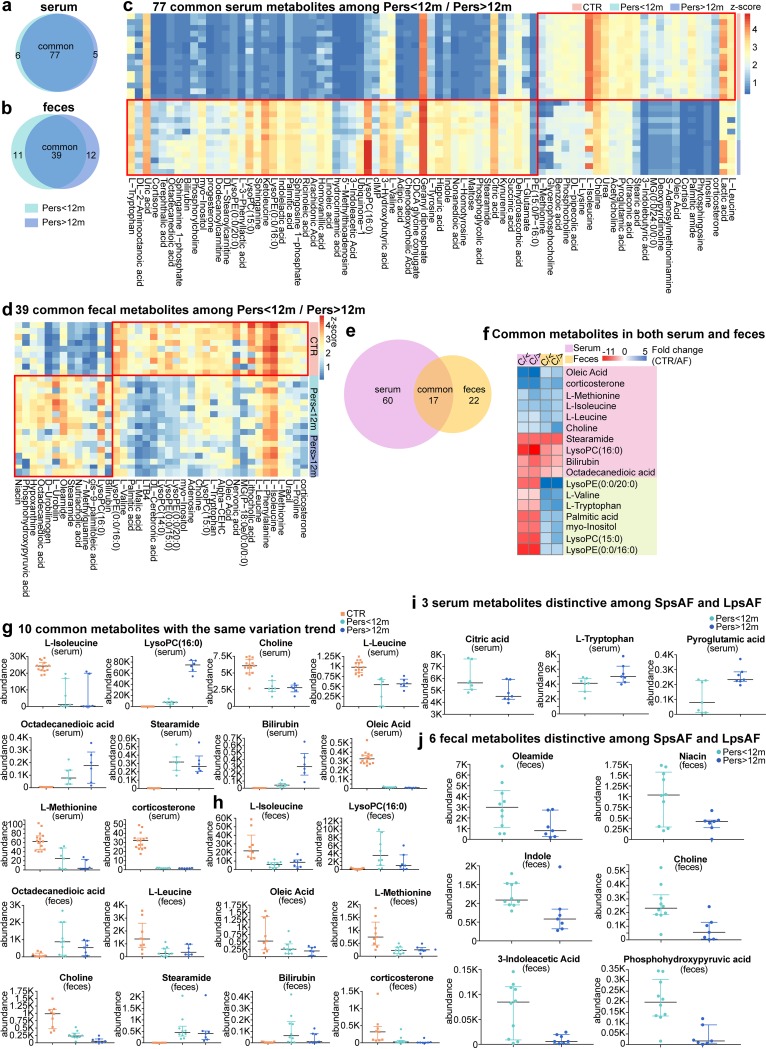
Alterations in serum and gut metabolomics of psAF. (a and b) Venn diagrams demonstrating the number of differential metabolites shared between Pers<12m (green) and Pers>12m (blue) compared with CTR. The overlap shows that there were 77 serum and 39 fecal metabolites concurrently identified in psAF of short or long duration. (c and d) Heat map of relative abundance of the 77 serum (c) and 39 fecal (d) common metabolites at the criterion of *P* value of <0.05 (*t* test). The abundance profiles are transformed into Z scores by subtracting the average abundance and dividing the standard deviation of all samples. Z score is negative (shown in blue) when the row abundance is lower than the mean and shown in red when the row abundance is higher than the mean. (e) Venn diagrams demonstrating the number of altered metabolites shared between serum (purple) and feces (yellow). The overlap shows that there were 17 endogenous compounds concurrently identified in both feces and serum. (f) Heat map of fold change (CTR/psAF) of 17 compounds which were altered in both serum and stool samples of AF patients. The fold change was transformed into t-scores, and t-score is negative (shown in red) when the compound showed an increased tendency in the Pers<12m or Pers>12m group. Compounds which increased or decreased simultaneously (*n* = 10) or individually (*n* = 7) in feces and serum are shown in pink and green, respectively. (g and h) Scatter plot of 10 metabolites simultaneously identified in both feces and serum. The dots indicate individual values of the subjects, and the horizontal lines from bottom to top represent 25th percentiles, medians, and 75th percentiles, respectively. (i and j) Scatter plot of 3 serum (i) and 6 fecal (j) distinctive metabolites between Pers<12m (green) and Pers>12m (blue). The dots indicate individual values of the subjects, and the horizontal lines from bottom to top represent 25th percentiles, medians, and 75th percentiles, respectively.

**FIG 7:**
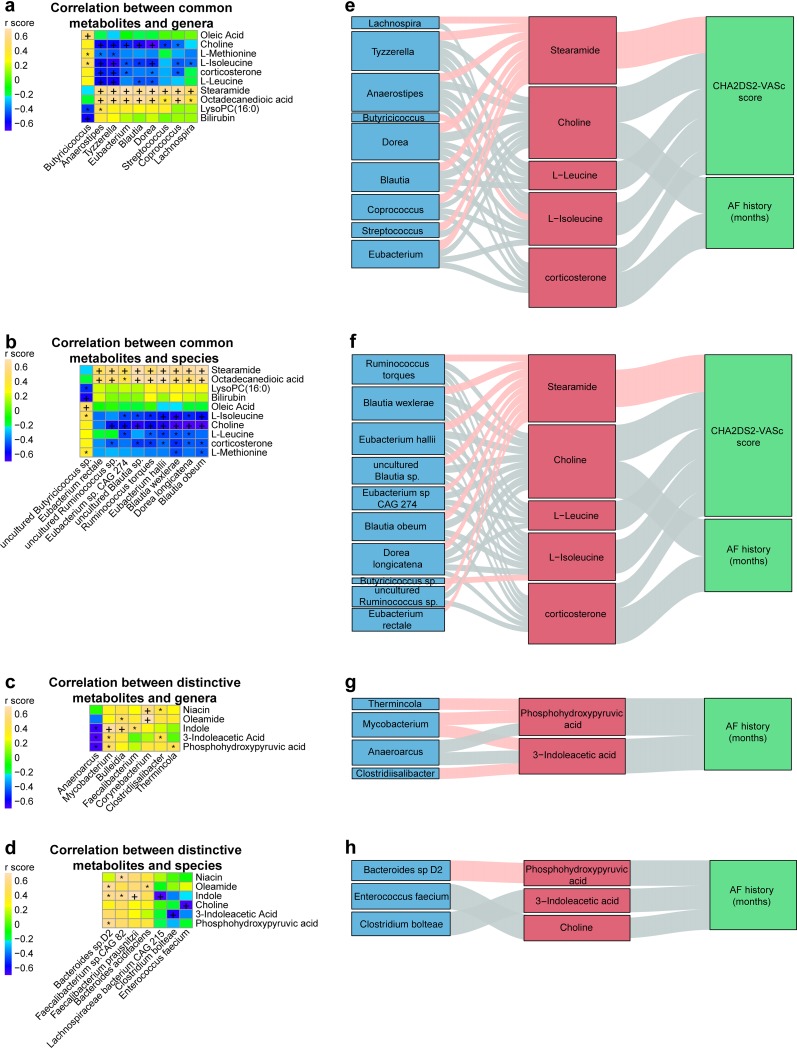
Correlation between dysbiotic GM and progression of psAF. (a and b) The relationship between 10 simultaneous metabolites and the top 10 common genera (a) and species (b). Considering that the abundance of fecal metabolites reflected the metabolites produced by GM, the fecal metabolomics data were used in Spearman correlation analysis. The heat map showed positive or negative correlation between the common genera (a) or species (b) and the metabolites. *, *P* < 0.05; +, *P* < 0.01. (c and d) The relationship between the distinctive metabolites in feces and the genera (c) or species (d). The color in the heat map indicated the r score of the spearman correlation. An r score >0 means positive correlation, and an r score <0 means negative correlation. *, *P* < 0.05; +, *P* < 0.01. In panels a to d, only metabolites and genera that reach a criterion of correlated with at least one taxon with *P* < 0.05 were shown. (e and f) Interrelationship between GM composition (common genera [e] and common species [f]), fecal metabolic profile, and AF phenotype. Visualization of the correlation network according to Spearman correlation analysis between the GM of the significant taxon and parameters of AF severity mediated by fecal metabolites. Red connections indicate a positive correlation (Spearman correlation test, false-discovery rate [FDR] < 0.05), while blue connections show correlations that were negative (Spearman correlation test, FDR < 0.05). (g and h) Interrelationship between GM composition (distinctive genera [g] and distinctive species [h]), fecal metabolic profile, and AF phenotype. Visualization of the correlation network according to Spearman correlation analysis between the GM of the significant taxon and parameters of AF severity mediated by fecal metabolites. Red connections indicate a positive correlation (Spearman correlation test, FDR < 0.05), while blue connections show correlations that were negative (Spearman correlation test, FDR < 0.05).

The PCA, the partial least-squares-discriminant analysis (PLS-DA), and the orthogonal PLS-DA (OPLS-DA) were plotted to reveal global metabolic changes between different groups of samples. For both the serum and fecal samples, a clear separation between Pers<12m and Pers>12m patients and controls was obtained in both ES^+^ and ES^−^ modes (Fig. S3 and S4a to n). Significantly differentially enriched metabolites were then identified on the basis of having a variable importance in the projection threshold of >1 and *P* < 0.05 and were further matched in the Metlin database. Overall, 77 altered serum metabolites and 39 altered stool metabolites were detected in both the Pers<12m and Pers>12m patients compared to controls (Fig. 6a and Fig. 7b). These metabolites comprised the majority of total metabolites differentially enriched in Pers<12m or Pers>12m compared with controls. Again, like our findings in microbial function modules, most of the altered metabolites exhibited analogous shifts in Pers<12m and Pers>12m (Fig. 6c and d), which implied similar metabolic patterns in psAF of short or long duration. Notably, 17 metabolites were altered in both the serum and stool samples of patients with psAF (Fig. 6e), 10 of which showed the same variation trend and were the focus of further investigation (Fig. 6f and Table S6). We identified compositional changes in Pers<12m and Pers>12m patients, with several enriched metabolites, including stearamide, octadecanedioic acid, and lysophosphatidylcholine (LysoPC) (16:0). It is important to note that octadecanedioic acid is a potent inducer of cardiac cell death and intracellular lipid accumulation (32). We identified six metabolites with significantly lower abundance in Pers<12m and Pers>12m, including oleic acid, choline, and some amino acids (Fig. 6g and h).

10.1128/mSystems.00422-19.3 FIG S3 Distinctive serum metabolic patterns between Pers<12m, Pers>12m, and control. (a and b) PCA plots based on the metabolic profiles in serum samples from control and psAF of short or long duration in ES^+^ (a) and ES^−^ (b). Orange squares represent CTR, green circles denote Pers<12m, and blue circles denote Pers>12m. The metabolic profiles in psAF of short or long duration were similar to each other but discriminative from controls under both ES^+^ and ES^−^ modes. (c and d) Partial least-squares discriminant analysis (PLS-DA) score plots based on the metabolic profiles in serum samples from control and Pers<12m group in ES^+^ (c) and ES^−^ (d). Orange squares represent CTR, and green circles denote Pers<12m. A clear separation between Pers<12m patients and controls was obtained under both ES^+^ and ES^−^ modes. (e and f) Score scatter plots of orthogonal PLS-DA (OPLS-SA) score plots based on the metabolic profiles in serum samples from control and Pers<12m groups in ES^+^ (e) and ES^−^ (f). Orange squares represent CTR, and green circles denote Pers<12m. A clear separation between Pers<12m patients and controls was obtained under both ES^+^ and ES^−^ modes. (g and h) Partial least-squares discriminant analysis (PLS-DA) score plots based on the metabolic profiles in serum samples from control and Pers>12m groups in ES^+^ (g) and ES^−^ (h). Orange squares represent CTR, and blue circles denote Pers>12m. A clear separation between Pers>12m patients and controls was obtained under both ES^+^ and ES^−^ modes. (i and j) Score scatter plots of orthogonal PLS-DA (OPLS-SA) score plots based on the metabolic profiles in serum samples from control and Pers>12m groups in ES^+^ (g) and ES^−^ (h). Orange squares represent CTR, and blue circles denote Pers>12m. A clear separation between Pers>12m patients and controls was obtained under both ES^+^ and ES^−^ modes. (k and l) Partial least-squares discriminant analysis (PLS-DA) score plots based on the metabolic profiles in serum samples from Pers<12m and Pers>12m groups in ES^+^ (k) and ES^−^ (l). Green circles represent Pers<12m, and blue circles denote Pers>12m. A clear separation between Pers<12m and Pers>12m was obtained under both ES^+^ and ES^−^ modes. (m and n) Score scatter plots of orthogonal PLS-DA (OPLS-SA) score plots based on the metabolic profiles in serum samples from Pers<12m and Pers>12m groups in ES^+^ (m) and ES^−^ (n). Green circles represent Pers<12m, and blue circles denote Pers>12m. A clear separation between Pers<12m and Pers>12m was obtained under both ES^+^ and ES^−^ modes. Download FIG S3, PDF file, 0.4 MB. Copyright © 2019 Zuo et al. 2019 Zuo et al. This content is distributed under the terms of the Creative Commons Attribution 4.0 International license.

10.1128/mSystems.00422-19.10 TABLE S6 Detailed information of 10 shared metabolites differently enriched across groups. Download Table S6, XLSX file, 0.02 MB. Copyright © 2019 Zuo et al. 2019 Zuo et al. This content is distributed under the terms of the Creative Commons Attribution 4.0 International license.

Moreover, three serum metabolites and six fecal metabolites were differentially identified between Pers<12m and Pers>12m (Fig. 6i and j). For example, the abundances of oleamide, niacin, indole, choline, 3-indoleacetic acid, and phosphohydroxypyruvic acid were decreased in the feces of Pers>12m patients, while l-tryptophan and pyroglutamic acid were elevated and citric acid was decreased in the serum of Pers>12m patients. These metabolic variations might be implicated in the arrhythmogenic substrate aggravation in the left atrium during the pathological progression of psAF.

### Correlation between dysbiotic GM and progression of psAF.

To explore the association between discrepant metabolites and disordered GM, we carried out a Spearman correlation analysis between the top 10 genera (Fig. 7a) or species (Fig. 7b) commonly altered in Pers<12m and Pers>12m patients and the 10 representative metabolites with similar variation tendencies. We found that 9 genera and 10 species were significantly associated with the 10 metabolites. Stearamide and octadecanedioic acid, previously associated with cardiovascular disease, were positively associated with Pers<12m- and Pers>12m-enriched genera such as *Eubacterium*, *Blautia*, and *Dorea* and species like uncultured *Ruminococcus* sp., Dorea longicatena, Blautia obeum, uncultured *Blautia* sp., Ruminococcus torques, Blautia wexlerae, Eubacterium hallii, and *Eubacterium* sp. CAG:274. Levels of Ruminococcus torques are higher in children with autism spectrum disorder with a reported functional gastrointestinal disorder (33). Subsequently, we assessed the metagenomics-based microbial associations between fecal metabolites and gut bacteria, where the gut microbiome could explain up to 46.66% of the variation in fecal metabolite choline in the current cohort. Some of the common metabolites showed very modest effects and could be explained jointly, where the explained variation was 15.45% for l-methionine, 22.84% for l-isoleucine, and 6.58% for bilirubin. The close relationship between microbes and metabolites indicates that these specific metabolites might be affected or produced, at least indirectly, by corresponding gut microbes, which requires further investigation. Furthermore, significant correlations were verified between distinctively altered bacteria and metabolites in Pers<12m and Pers>12m (Fig. 7c and d), including a relationship between indole and *Faecalibacterium* (genus) or Faecalibacterium prausnitzii or *Faecalibacterium* sp. CAG 82 (species) and the association between 3-indoleacetic acid and *Clostridiisalibacter* (genus) or Clostridium bolteae (species). Based on the significant correlation between the distinguishing metabolic features in psAF and the disordered GM, it is possible that the GM dysbiosis causes a decrease in some cardiovascular system-protective metabolites and/or an excess in harmful substances and thereby contributes to the progression of psAF.

To further explore the relationship of disordered GM in psAF of short or long duration mediated by metabolites, we carried out a Spearman correlation analysis between the top 10 common gut microbes, top 10 common fecal metabolites, distinctive gut microbes, and distinctive fecal metabolites. The clinical AF scores of nine genera and 10 species were significantly correlated with six fecal metabolites, and these metabolites were further correlated with the AF duration and CHA2DS2-VASc score (congestive heart failure, HTN, age ≥75 years, diabetes mellitus, stroke/transient ischemic attack, vascular disease, age 65 to 74 years, sex category of female), which represents the severity of atrial remodeling in psAF patients (34–37). Some commonly enriched genera in psAF, including *Tyzzerella*, *Anaerostipes*, *Dorea*, and *Eubacterium*, were positively correlated with stearamide, a metabolite enriched in Pers<12m and Pers>12m, which was positively linked with CHA2DS2-VASc score (Fig. 7e). Meanwhile, these commonly enriched genera in psAF were negatively correlated with choline, a metabolite decreased in Pers<12m and Pers>12m, which was negatively linked with CHA2DS2-VASc score (Fig. 7e). A similar trend was identified at the species level (Fig. 7f). Some species enriched in Pers<12m and Pers>12m patients were positively correlated with stearamide and CHA2DS2-VASc score, whereas species reduced in Pers<12m and Pers>12m patients were negatively correlated with CHA2DS2-VASc score and AF duration through certain metabolites. In particular, genera including *Blautia* and *Dorea* and species including Blautia wexlerae, Blautia obeum, and Dorea longicatena were closely related to the progression of psAF, metabolites, and clinical psAF parameters.

We also constructed the interaction network for distinctive microbes and metabolites between Pers<12m and Pers>12m and AF-associated parameters. Notably, the abundance of choline was significantly decreased with prolonged AF duration, and choline was negatively associated with Enterococcus faecium (Fig. 7g). Moreover, phosphohydroxypyruvic acid and 3-indoleacetic acid, the metabolites decreased in Pers>12m, were negatively correlated with psAF duration and the Pers>12m-enriched genus *Anaeroarcus* but positively correlated with the Pers>12m decreased genus *Mycobacterium*. These matched interactions were further identified at the species level (Fig. 7h). These connected alterations in microbes and metabolites indicate that these microbes might participate in the progression of psAF by interacting with various host metabolites.

## DISCUSSION

Although our recent study identified the disordered GM profiles in AF patients, studies characterizing the GM features in subtypes of AF patients are still lacking (22). In the present study, we acquired new evidence describing the characteristics of a dysbiotic GM and metabolism according to psAF duration. We used metagenomic and metabolomic data to characterize psAF patients classified as Pers<12m and Pers>12m. We identified similarly increased GM diversity in psAF of short and of long duration and found a cluster of bacteria that were enriched in Pers<12m and Pers>12m. We further observed a simultaneous disturbance in GM function and metabolic alterations and linked the simultaneous alterations of the gut microbiome and metabolome to AF progression, which indicates a crucial role of the GM in AF persistence through interacting with the host metabolites. These findings are fundamental for further studies aiming to explore the precise contribution of the GM to AF development.

One of the most important findings from the present study is that GM dysbiosis has already occurred in Pers<12m and is maintained in Pers>12m. Studies regarding the correlation between gut microbiome and progression of disease have shown a similar phenomenon. Our previous study identified that the microbiome features in pre-HTN individuals were quite similar to those in patients with HTN (12). Furthermore, a metagenomic analysis of chronic heart failure patients also revealed that patients exhibited similar changes in GM composition and metabolic features, regardless of whether the causation was ischemic cardiomyopathy or dilated cardiomyopathy (23). Consistently, a recent study confirmed that a significantly altered GM has already developed in individuals at preclinical stages of rheumatoid arthritis (38). These intriguing results imply a possible causal role of discrepant GM in contributing to the pathogenesis, development, and aggravation of diseases indirectly, which is consistent with conclusions drawn by fecal transplantation experiments. Similarly, the common profiles of the GM and metabolism that we found in Pers<12m and Pers>12m in the current study might be correlated with or even participate in the onset of AF.

Notably, besides the similarities shared by Pers<12m and Pers>12m, the disparity in psAF of short or long duration was also revealed, which may be linked to the maintenance of AF progression. For example, Clostridium bolteae (a species enriched in Pers>12m) is a bacterium that has been shown to be overabundant in the intestinal tract of autistic children suffering from gastric intestinal ailments, which produces a conserved specific capsular polysaccharide (39). Faecalibacterium prausnitzii (a species decreased in Pers>12m), is an abundant obligate anaerobe that colonizes during weaning and is thought to maintain colonic health throughout life. This species may be a useful potential biomarker to assist in ulcerative colitis and Crohn’s disease discrimination (40). It has been previously revealed that Faecalibacterium prausnitzii induced Toll-like receptor 2 (TLR2) activation, which is linked to enhanced tight junction formation, while its role in enhancing epithelial barrier integrity requires further investigation (41). Meanwhile, Faecalibacterium prausnitzii treatment improved hepatic health and reduced adipose tissue inflammation in mice fed a high-fat diet (42). The alterations of the GM were coupled with metabolic phenotype, which was influenced by interaction with the intestinal bacteria. A 20-year cohort study following >74,000 participants revealed that oleic acid (decreased in Pers<12m and Pers>12m compared with the control) consumption significantly reduced the risk for developing cardiovascular diseases. Oleic acid prevents coronary heart disease by suppressing oxidative stress, mitigating cardiomyocyte cell damage. In addition, octadecanedioic acid-induced lipotoxicity, as mentioned above, could be antagonized by the unsaturated fatty acid oleic acid (32). Niacin (decreased in Pers>12m) is a potent high-density lipoprotein cholesterol-raising drug and has been proposed as an attractive approach to reduce cardiac events in patients with or at risk of atherosclerotic cardiovascular disease (43). Niacin has been used for primary and secondary coronary heart disease prevention for over 40 years. Until recently, clinical trials incorporating niacin as part of an intervention strategy consistently demonstrated reduction in clinical events and lesion improvement (44). Furthermore, recent studies have demonstrated that choline (decreased in Pers>12m), an essential dietary nutrient for humans, is required for the synthesis of the neurotransmitter acetylcholine, the methyl group donor betaine, and phospholipids. Therefore, choline is involved in a broad range of critical physiological functions across all stages of the life cycle (45). Notably, a previous study concluded that choline prevents angiotensin II (Ang II)-induced cardiac hypertrophy through inhibition of reactive oxygen species (ROS)-mediated p38 mitogen-activated protein kinase (MAPK) activation as well as regulation of the Ca^2+^-mediated calcineurin signal transduction pathway (46). Therefore, it is possible that a dysbiotic GM and subsequent altered host metabolism may be an early modulator of AF development and might be regarded as a target for future preventive interventions in individuals at risk of AF, before the progression of AF.

It is well known that AF is a clinically heterogeneous arrhythmia (47). In most cases, AF progresses from low to heavy burden and from short, infrequent episodes to longer and more frequent attacks. Many AF patients do not receive therapy until the AF burden becomes heavier. However, longer AF duration is accompanied by remarkable and irreversible atrial remodeling and predicts a low sinus rhythm maintenance rate after therapy for rhythm controlling (48). AF-induced atrial remodeling enhances the vulnerability of the heart to AF induction and maintenance, with alterations in atrial refractoriness, changes in cellular calcium homeostasis, autonomic activation, and after-depolarizations, which contribute to triggered activity and AF initiation. Further, structural remodeling dominated by atrial fibrosis leads to local conduction disturbances and conduction block, which facilitate reentry and AF maintenance. This autoreinforcing property of AF is often summarized as “AF begets AF” (49, 50). The AF termination rate declines in patients progressing to longstanding psAF. Therefore, identifying early alterations in patients with AF and establishing earlier intervention strategies offer a precious opportunity to halt the progressive pathoelectrophysiological and anatomical changes associated with AF (51, 52). Our current findings provide further evidence and support the significance of early intervention for AF.

AF is a disease with dynamic progression. Based on the presentation, duration, and spontaneous termination of AF episodes, AF is traditionally distinguished as paroxysmal, persistent, and longstanding persistent AF (10). To reduce the heterogeneity among participants, this study focused on patients with psAF, and future work comparing paroxysmal and persistent AF patients is still needed. Moreover, by continuous feces sample collection from the patient cohort during a follow-up period, a dynamic observation of the dysbiotic GM pattern might provide stronger evidence. Finally, the number of participants was relatively small, and further studies with an expanded sample size and systemic follow-up are still needed.

### Conclusions.

The present study provides a comprehensive description of the dysbiotic GM and host metabolic profiles in patients with Pers<12m and Pers>12m and concludes that psAF of short or long duration shared most of the common features of GM dysbiosis. However, some distinctive and progressive alterations in GM and metabolic structure which may contribute to the progression of psAF were identified. The occurrence of GM dysbiosis was identified in the early stages of AF, and intervention strategies targeting dysbiotic GM to postpone AF progression may be clinically valuable.

## MATERIALS AND METHODS

### Study cohort.

Twenty nonvalvular persistent AF (psAF) patients and 20 controls (CTR) were included from our previous work on GM in AF (22). Participants with a history of heart failure, coronary heart disease, structural heart disease, comorbidities (inflammatory bowel diseases, irritable bowel syndrome, autoimmune diseases, liver diseases, renal diseases, or cancer), or use of antibiotics or probiotics in the last month were excluded. Clinical baseline characteristics were obtained via face-to-face surveys and medical records. The research protocol was approved by the ethics committee of Beijing Chaoyang Hospital and Kailuan General Hospital. All of the participants signed informed consent forms.

The 20 psAF patients were divided into two groups based on AF duration and manifestation of electrocardiogram. Patients with AF lasting longer than 7 days but less than 1 year were classified as Pers<12m, while patients with psAF for more than 1 year were classified as Pers>12m (10). The study was approved by the ethics committee of Beijing Chaoyang Hospital and Kailuan General Hospital. All participants signed informed consents.

### Analyses of GM based on metagenome.

The whole-metagenome shotgun sequencing data of the 40 feces samples in this cohort were obtained as part of a previously published study (22). The analyses of metagenomic sequencing, gene catalogue construction, gene prediction, taxonomic assignment, taxonomic and functional annotation, abundance profiling, and analysis of enterotype were all performed as we previously described (12, 22). Paired-end metagenomic sequencing was performed on the Illumina platform (insert size 300 bp, read length 150 bp). After quality control, the reads aligned with the human genome (alignment with Short Oligonucleotide Analysis Package 2 [SOAP2], version 2.21, parameters: −s 135, −l 30, −v 7, −m 200, −x 400) were removed. The assembly of reads was executed by SOAP *de novo* (version 2.04, parameters: −d 1 −M 3 −R −u −F), and the clean data were mapped against scaffolds by SOAP2 (version 2.21, parameters: −m 200 −x 400 −s 119). Subsequently, gene prediction from the assembled contigs was performed by MetaGeneMark (prokaryotic GeneMark, hidden Markov model version 2.10). A nonredundant gene catalogue was constructed with Cluster Database at High Identity with Tolerance (CD-HIT, version 4.5.8, parameters: −G 0 −aS 0.9 −g 1 −d 0 −c 0.95). Reads were realigned to the gene catalogue with SOAP2 using parameters to determine the abundance of genes. The gene abundance was calculated by counting the number of reads and normalized by gene length. Then, to assess the taxonomic assignment, genes were aligned to the integrated nr database by DIAMOND (version 0.7.9.58, default parameters except that −k 50 −sensitive −e 0.00001). Significant matches for each gene, defined by E values of ≤10× E value of the top hit, were determined, and the retained matches were used to distinguish taxonomic groups. The taxonomical level of each gene was identified according to the lowest common ancestor-based algorithm implemented with MEGAN (MEtaGenome ANalyzer), and the abundance of a taxonomic group was calculated according to the sum abundance of genes annotated to a feature. To assess the function of gut bacteria, all genes in the catalogue were aligned to the KEGG database (release 73.1, with animal and plant genes removed) by DIAMOND (version 0.7.9.58, default parameters except for –k 50 –sensitive –e 0.00001), and each protein was assigned to the KEGG database using the highest-scoring annotated hits containing ≥1 high-scoring segment pair scoring >60 hits. Through summing the abundance of genes annotated to the same feature, the abundance of the KEGG module was calculated.

### Analyses of GM based on metabolome.

In the 40 subjects enrolled in the current study, metabolomic data from 29 serum and 26 feces samples based on liquid chromatography-mass spectrometry (LC-MS) were available from our previous study. Methods of feature extraction, data normalization, and identification of compounds were carried out as previously described. The metabolomic data were prepared for feature extraction and preprocessed with Compound Discoverer 2.0 software (Thermo Fisher Scientific). Normalized data were edited into a two-dimensional (2D) data matrix by Excel 2010 software, using retention time (RT), compound molecular weight (compMW), observations (samples), and peak areas. Multivariate analysis was then performed by SIMCA-P software (Umetrics AB, Umea, Sweden). The exact molecular mass, parts per million (ppm) (<25), and tandem mass spectrometry value of these compounds were selected to identify metabolites associated with the featured peak in the Metlin database. In addition, the mass spectra were compared, and the score value that indicated a matching rate was calculated by Compound Discoverer 2.0 software (Thermo Fisher Scientific) with a maximum of 100. When the metabolites were detected in both ES^+^ and ES^−^, the data in the mode with the lower *P* value were retained for further analysis.

### Statistical analysis.

Quantitative data with normal distributions are presented as mean and standard deviation, while quantitative data with nonnormal distributions are presented as median (first quartile, third quartile), and the *t* test or Wilcoxon rank sum test was performed for between-group comparisons. Qualitative data are presented as a percentage, and the χ^2^ test was used for between-group comparisons.

The alpha diversity, including Pielou evenness, Shannon index, and Chao richness, at the genus and species level was calculated with R software (version 3.3.3, package vegan). The beta diversity, including principal-component analysis (PCA), was performed using the FactoMineR package in R software (version 3.3.3), while principal-coordinate analysis (PCoA) was performed using the vegan and ape package in R software (version 3.3.3), and nonmetric dimensional scaling (NMDS) was performed by using the vegan package in R software (version 3.3.3); all plots were visualized by the package ggplot2 in R software (version 3.3.3). The partial least-squares discriminant analysis (PLS-DA) and the orthogonal partial least-squares discriminant analysis (OPLS-DA) were carried out using the SIMCA-P software to cluster sample plots across groups. Differential abundance of genes, genera, species, and KEGG modules was determined using the Wilcoxon rank sum test, and *P* values were corrected for multiple testing with the Benjamini and Hochberg method. Enterotyping was performed as previously described (53). Briefly, all samples were analyzed by the partitioning-around-medoids clustering method based on the Jensen-Shannon distance from the genus profile, and the optimal number of clusters was estimated using the Calinski-Harabasz (CH) index. Only genera identified in at least 10% of samples were considered.

The Spearman correlation of metabolic and microbiome abundances was used to identify microbiome-metabolome association. In order to estimate the variation of fecal metabolites explained by microbial factors, first, we used the caret package in R software (version 3.3.3) to preprocess the predictor data, which included all identified species and genus, and then, the LASSO shrinkage model from the R package ‘glmnet’ (v.2.0.18) was used to estimate the proportion of variation in fecal metabolism explained by microbial composition. Spearman correlations between genera/species, fecal metabolites, and clinical AF parameters were calculated by R, and the visual presentation of Sankey was performed via the R package riverplot (version 0.6). All statistical tests were 2 sided, and *P* < 0.05 was regarded as significant.

### Data availability.

The data supporting the results of this article have been deposited in the EMBL European Nucleotide Archive (ENA) under the BioProject accession code PRJEB28384. The metabolomics data are available at the NIH Common Fund’s Data Repository and Coordinating Center website with Metabolomics Workbench Study identifiers ST001168 (for fecal metabolomic analyses) and ST001169 (for serum metabolomic analyses).

10.1128/mSystems.00422-19.4 FIG S4 Distinctive fecal metabolic patterns between Pers<12m, Pers>12m, and control. (a and b) PCA plots based on the metabolic profiles in fecal samples from control and psAF of short or long duration in ES^+^ (a) and ES^−^ (b). Orange squares represent CTR, green circles denote Pers<12m, and blue circles denote Pers>12m. The metabolic profiles in psAF of short or long duration were similar to each other but discriminative from controls under both ES^+^ and ES^−^ modes. (c and d) Partial least-squares discriminant analysis (PLS-DA) score plots based on the metabolic profiles in fecal samples from control and Pers<12m groups in ES^+^ (c) and ES^−^ (d). Orange squares represent CTR, and green circles denote Pers<12m. A clear separation between Pers<12m patients and controls was obtained under both ES^+^ and ES^−^ modes. (e and f) Score scatter plots of orthogonal PLS-DA (OPLS-SA) score plots based on the metabolic profiles in fecal samples from control and Pers<12m groups in ES^+^ (e) and ES^−^ (f). Orange squares represent CTR, and green circles denote Pers<12m. A clear separation between Pers<12m patients and controls was obtained under both ES^+^ and ES^−^ modes. (g and h) Partial least-squares discriminant analysis (PLS-DA) score plots based on the metabolic profiles in fecal samples from control and Pers>12m groups in ES^+^ (g) and ES^−^ (h). Orange squares represent CTR, and blue circles denote Pers>12m. A clear separation between Pers>12m patients and controls was obtained under both ES^+^ and ES^−^ modes. (i and j) Score scatter plots of orthogonal PLS-DA (OPLS-SA) score plots based on the metabolic profiles in fecal samples from control and Pers>12m groups in ES^+^ (i) and ES^−^ (j). Orange squares represent CTR, and blue circles denote Pers>12m. A clear separation between Pers>12m patients and controls was obtained under both ES^+^ and ES^−^ modes. (k and l) Partial least-squares discriminant analysis (PLS-DA) score plots based on the metabolic profiles in fecal samples from Pers<12m and Pers>12m groups in ES^+^ (k) and ES^−^ (l). Green circles represent Pers<12m, and blue circles denote Pers>12m. A clear separation between Pers<12m and Pers>12m was obtained under both ES^+^ and ES^−^ modes. (m and n) Score scatter plots of orthogonal PLS-DA (OPLS-SA) score plots based on the metabolic profiles in fecal samples from Pers<12m and Pers>12m groups in ES^+^ (m) and ES^−^ (n). Green circles represent Pers<12m, and blue circles denote Pers>12m. A clear separation between Pers<12m and Pers>12m was obtained under both ES^+^ and ES^−^ modes. Download FIG S4, PDF file, 0.4 MB. Copyright © 2019 Zuo et al. 2019 Zuo et al. This content is distributed under the terms of the Creative Commons Attribution 4.0 International license.
