# Supplementary figures and images for: Duration of Persistent Atrial Fibrillation Is Associated with Alterations in Human Gut Microbiota and Metabolic Phenotypes
Source: mSystems. 2019 Dec 10;4(6):e00422-19. doi: 10.1128/mSystems.00422-19 (PMC6906738; doi:10.1128/mSystems.00422-19)

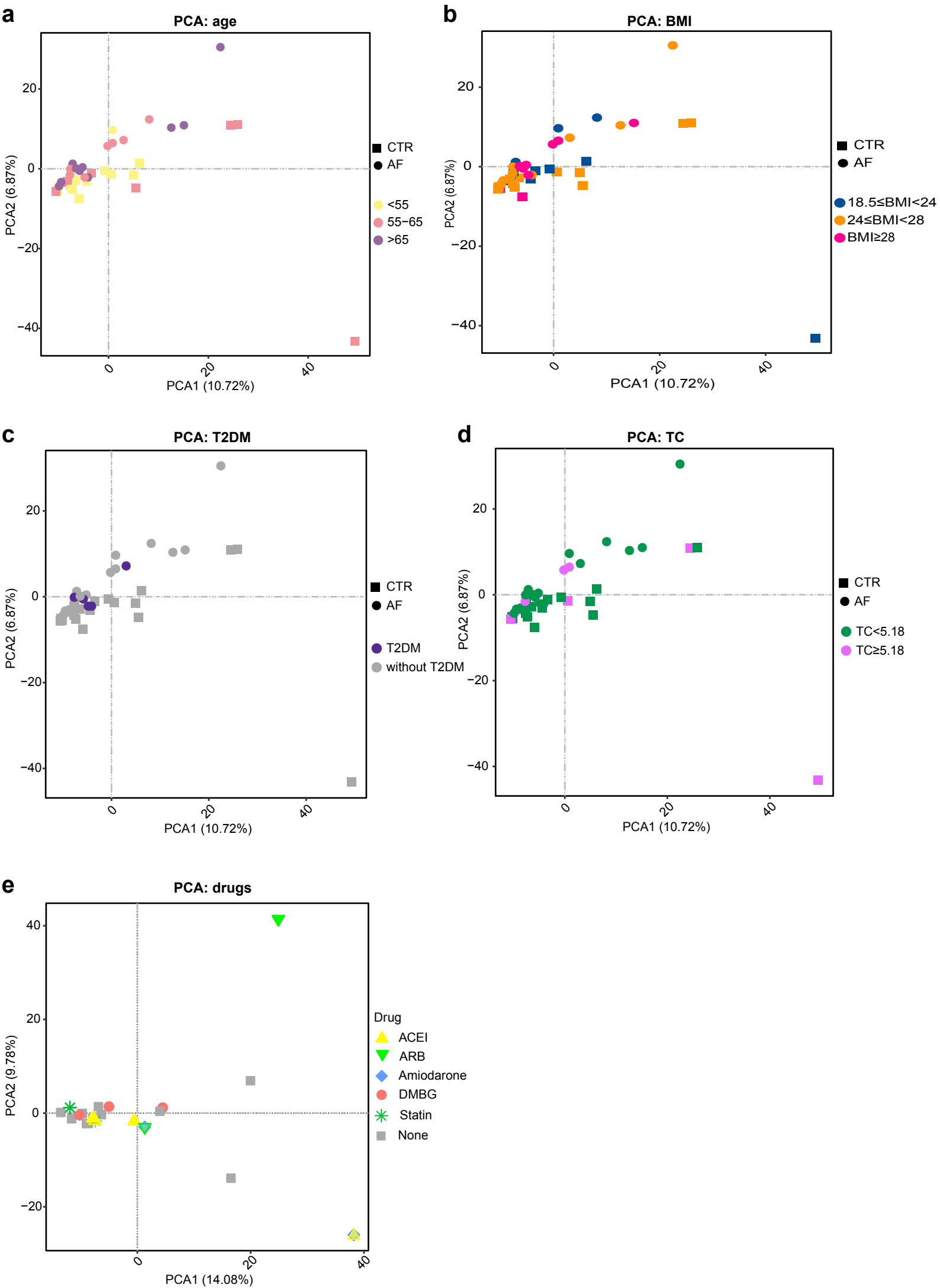

Supplement: FIG S1 [file mSystems.00422-19-sf001.pdf]

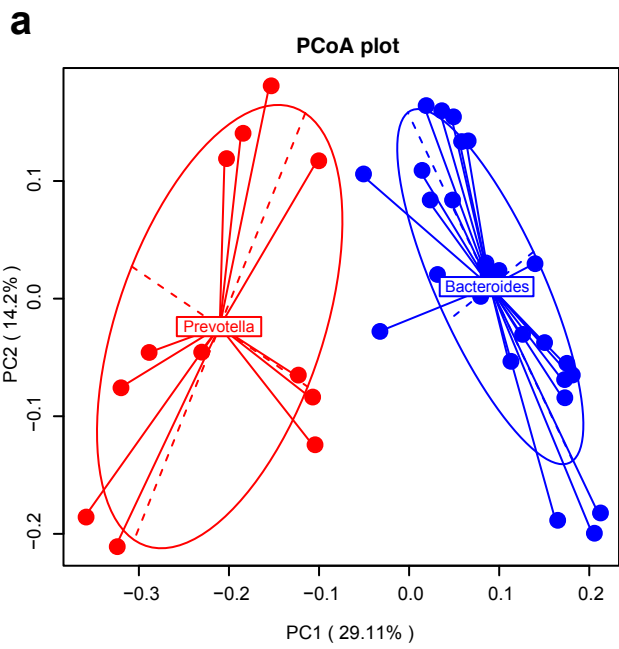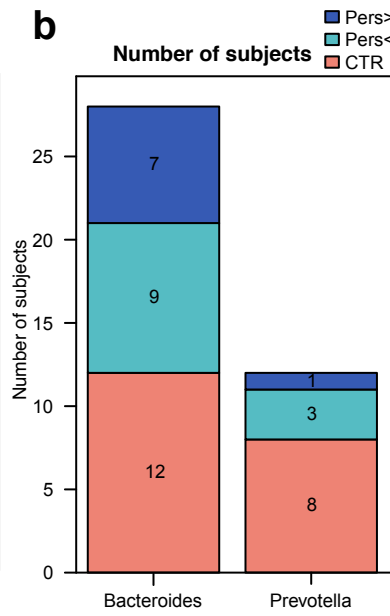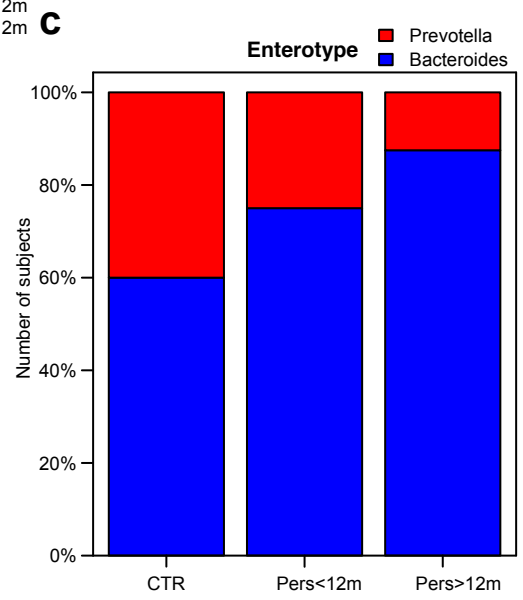

Supplement: FIG S2 [file mSystems.00422-19-sf002.pdf]

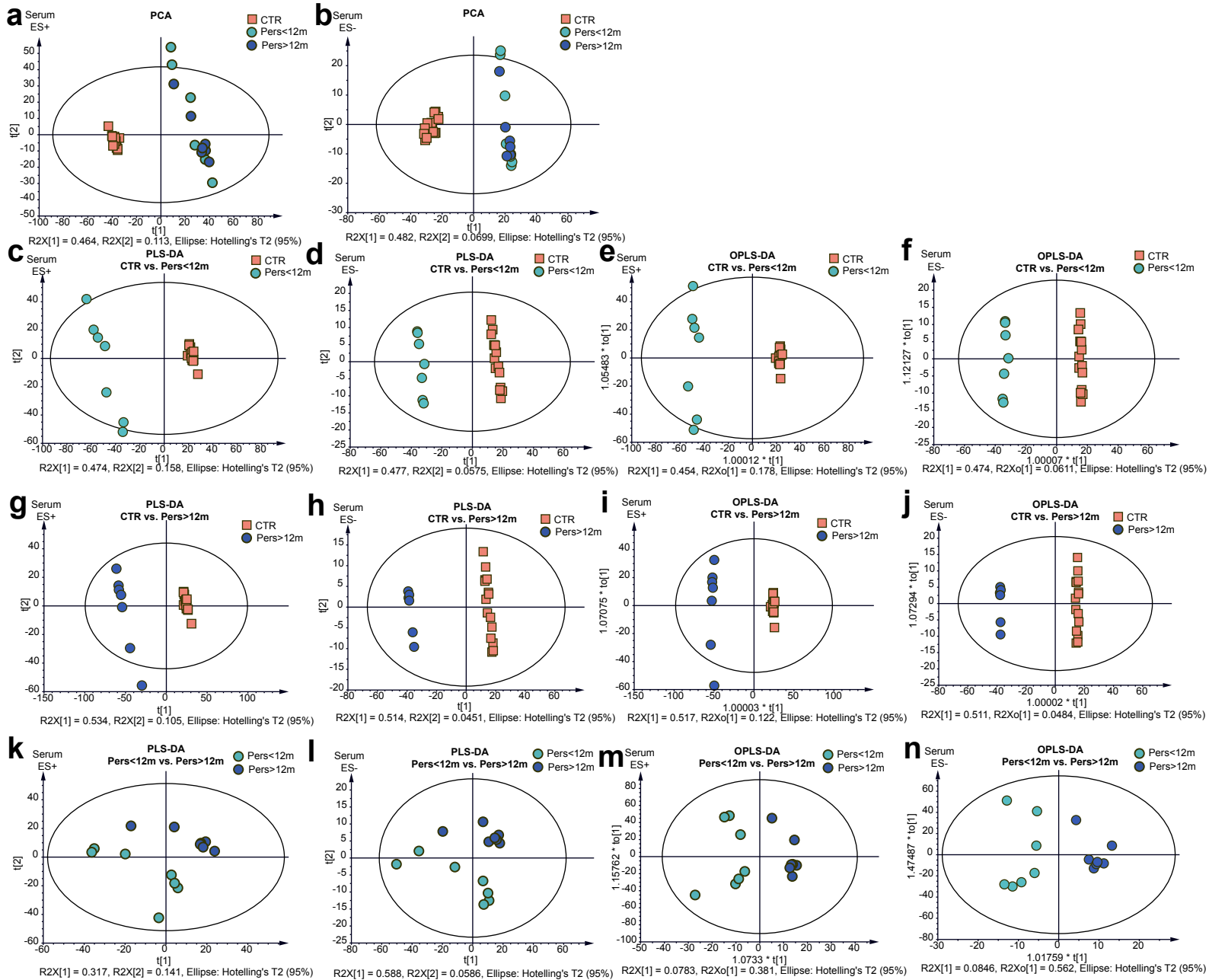

Supplement: FIG S3 [file mSystems.00422-19-sf003.pdf]

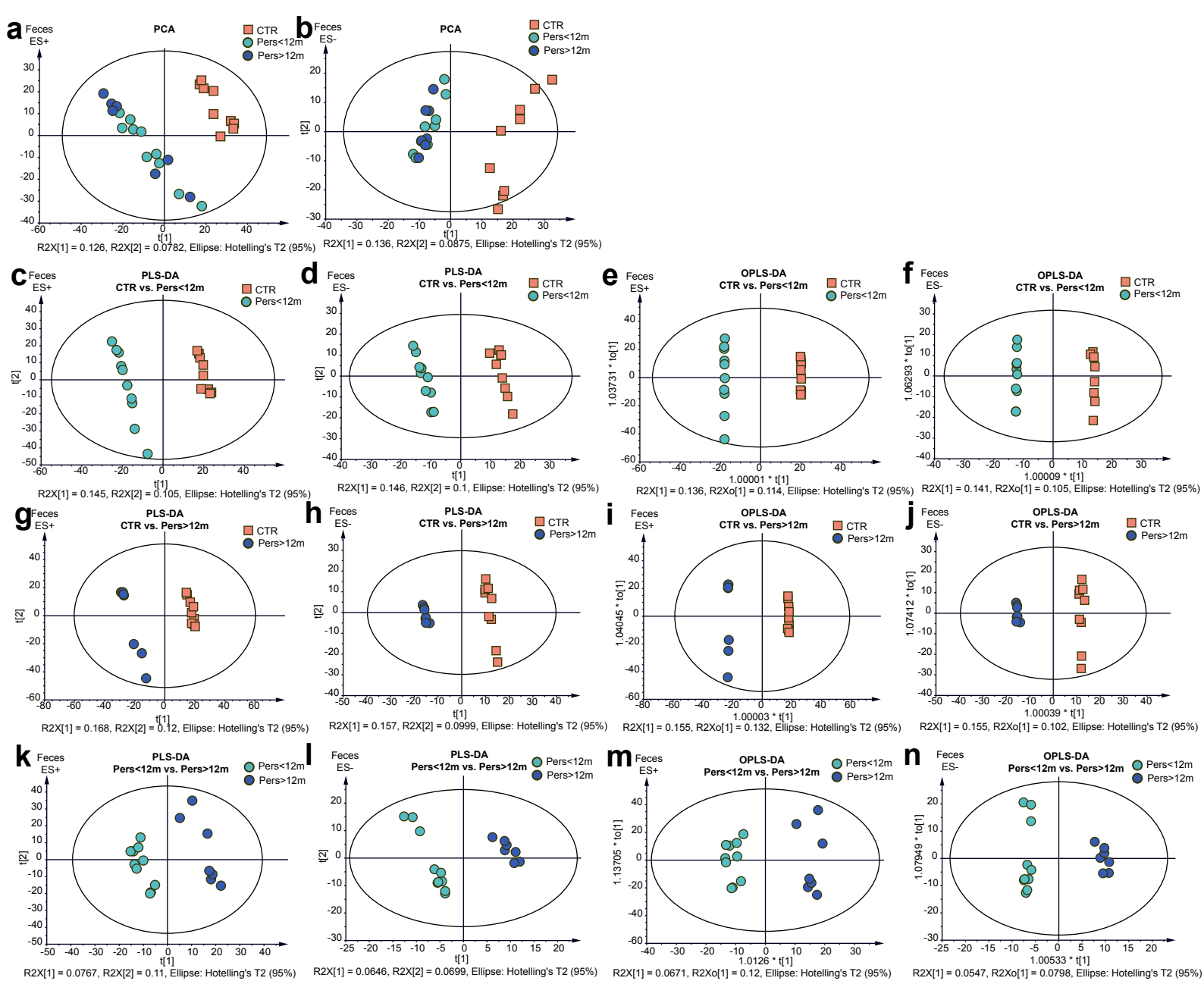

Supplement: FIG S4 [file mSystems.00422-19-sf004.pdf]
