# Supplementary material for: Duration of Persistent Atrial Fibrillation Is Associated with Alterations in Human Gut Microbiota and Metabolic Phenotypes
Source: mSystems. 2019 Dec 10;4(6):e00422-19. doi: 10.1128/mSystems.00422-19 (PMC6906738; doi:10.1128/mSystems.00422-19)
Supplement: TABLE S5 [file mSystems.00422-19-st005.docx]

| **Serum** | **CTR** | **Pers<12m** | **Pers>12m** | **P value**  **(CTR vs. Pers<12m)** | **P value**  **(CTR vs. Pers>12m)** | **P value**  **(Pers<12m vs. Pers>12m)** |
| --- | --- | --- | --- | --- | --- | --- |
| **Number** | 15 | 7 | 7 | / | / | / |
| **Age, years** | 52 (49, 55) | 68 (56, 77) | 68 (62, 72) | 0.011 | <0.001 | 0.805 |
| **Male/ Female** | 13/2 | 5/2 | 5/2 | 0.581 | 0.581 | 1.000 |
| **BMI** | 24.50 (20.52, 27.47) | 26.71 (23.24, 31.11) | 27.61 (25, 34.06) | 0.237 | 0.047 | 0.456 |
| **HTN** | 9 | 4 | 6 | 0.945 | 0.368 | 0.383 |
| **DM** | 0 | 2 | 2 | 0.298 | 0.298 | 1.000 |
| **TC** | 4.73 (4.47, 5.28) | 4.29 (3.09, 4.71) | 4.28 (3.50, 5.25) | 0.032 | 0.142 | 0.535 |
| **FBG** | 5.3 (4.98, 5.73) | 5.15 (4.58, 7.21) | 4.68 (4.45, 5.43) | 0.938 | 0.097 | 0.383 |
| **Creatinine** | 63 (57, 71) | 72.3 (66.3, 77.7) | 69.5 (59, 77.3) | 0.162 | 0.407 | 0.805 |
| **TBil** | 16.8 (12.72, 23.33) | 21.9 (13.3, 25.3) | 14.4 (12.8, 22.8) | 0.443 | 0.913 | 0.383 |
| **ALT** | 22 (12, 27) | 16 (10, 28) | 25 (12, 49) | 0.581 | 0.581 | 0.318 |

Table S5. Clinical characteristics of participants in serum and fecal metabolism.

| **Fecal** | **CTR** | **Pers<12m** | **Pers>12m** | **P value**  **(CTR vs. Pers<12m)** | **P value**  **(CTR vs. Pers>12m)** | **P value**  **(Pers<12m vs. Pers>12m)** |
| --- | --- | --- | --- | --- | --- | --- |
| **Number** | 9 | 10 | 7 | / | / | / |
| **Age, years** | 51 (48, 53.5) | 68.5 (57.5, 74) | 68 (62, 72) | 0.003 | <0.001 | 0.74 |
| **Male/ Female** | 9/0 | 6/4 | 5/2 | 0.156 | 0.351 | 0.74 |
| **BMI** | 24.49 (21.36, 27.35) | 24.71 (22.55, 28.08) | 27.61 (25, 34.06) | 0.661 | 0.071 | 0.133 |
| **HTN** | 7 | 4 | 6 | 0.182 | 0.837 | 0.133 |
| **DM** | 0 | 3 | 2 | 0.278 | 0.351 | 0.962 |
| **TC** | 4.52 (4.25, 4.92) | 3.92 (3.35, 4.61) | 4.28 (3.5, 5.25) | 0.043 | 0.408 | 0.536 |
| **FBG** | 5.39 (4.63, 5.94) | 5.14 (4.34, 6.51) | 4.68 (4.45, 5.43) | 0.829 | 0.189 | 0.417 |
| **Creatinine** | 63 (57.5, 69) | 71.45 (65.63, 80.05) | 69.5 (59, 77.3) | 0.028 | 0.21 | 0.74 |
| **TBil** | 19.1 (14.2, 24.55) | 21.45 (13.30, 24.7) | 14.4 (12.8, 22.8) | 0.968 | 0.536 | 0.417 |
| **ALT** | 22 (12, 26.5) | 21 (11.5, 28) | 25 (12, 49) | 0.661 | 0.47 | 0.601 |

Abbreviations: CTR, control; psAF, persistent atrial fibrillation; Pers<12m, persistent atrial fibrillation < 12 months; Pers>12m, persistent atrial fibrillation > 12 months; BMI, body mass index; HTN, hypertension; DM, diabetes mellitus; TC, total cholesterol; FBG, fasting blood glucose; TBil, total bilirubin; ALT, glutamic-pyruvic transaminase; IQR, interquartile range; Data are presented as mean± SD, or median (IQR), as appropriate.
